# Reliable, Fast and Stable Contrast Response Function Estimation

**DOI:** 10.3390/vision6040062

**Published:** 2022-10-17

**Authors:** Nelson Cortes, Marc Demers, Visou Ady, Lamyae Ikan, Christian Casanova

**Affiliations:** Visual Neuroscience Laboratory, School of Optometry, Université de Montréal, Montreal, QC H3C 3J7, Canada

**Keywords:** statistical curve fitting, contrast response function, Naka Rushton equation, visual cortical neurons

## Abstract

A study was conducted to determine stable cortical contrast response functions (CRFs) accurately and repeatedly in the shortest possible experimentation time. The method consisted of searching for experimental temporal aspects (number and duration of trials and number and distribution of contrasts used) with a model based on inhomogeneous Poisson spike trains to varying contrast levels. The set of values providing both short experimental duration and maximizing fit of the CRFs were saved, and then tested on cats’ visual cortical neurons. Our analysis revealed that 4 sets of parameters with less or equal to 6 experimental visual contrasts satisfied our premise of obtaining good CRFs’ performance in a short recording period, in which the number of trials seems to be the experimental condition that stabilizes the fit.

## 1. Introduction

Recent technological advances yielded an exponential increase in the number and size of the biological data simultaneously recorded [1]. At first sight, neuroscientists would benefit from these massive amounts of data to better understand brain functions [2], but new considerations have to be taken when experiments are done [2]. For example, if individual responses within a large neural population were optimized as a function of external visual stimuli, the amount of time required to record such data would be enormous. One solution would be to perform adaptive methods, such as those used in psychophysics [3], and acquire the average performance of neural populations to visual stimuli. Another approach would be to know a priori the optimal visual responses of this group of neurons and thus use these already optimized variables to reduce the recording time required.

An example of technological advancement is the development of multi-channel electrodes, which allow the recording of the activity of hundreds if not thousands of neurons simultaneously [4]. When recording with a single electrode, researchers can extract rapidly and efficiently neuronal properties from raw signals. However, the use of a multi-electrode array poses new challenges for neuroscientists regarding the optimization of stimulation protocols and the subsequent analyses, increasingly so if it is performed online. Therefore, new tools for extracting specific parameters from experimental data are required to exploit and maximize the recording of a large neural data set.

One of the most investigated properties of neurons in the visual system is their response to stimulus contrast changes leading to the contrast response function (CRF) [5]. A sigmoidal shape characterizes the CRF, increasing nonlinearly as a power function at low contrast levels, approximately linear at middle levels, and saturating at high contrasts. The CRF can be fitted by the Naka-Rushton Equation (NRE) [6]. Assessing the CRF of a large pool of neurons is essential when one wants to characterize a given brain area’s functional properties but remains challenging in time and accuracy.

**Figure 1:**
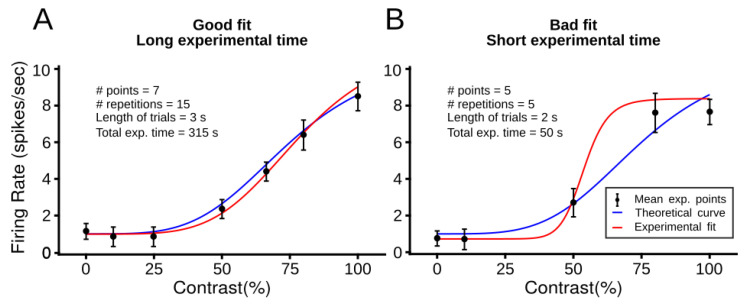
Trade-off between the fit performance and the recording time to obtain reliable CRFs. While the performance of the fit is measured by the comparison between theoretical and experimental curves, the recording time comes from the multiplication of experimental conditions (# points, # repetitions, length of trials). Theoretical curves (blue lines) are obtained directly by the NRE to a given set of parameters. Experimental curves (red lines) are generated by the fit of the average firing rate to visual contrast stimuli (black dots). Two representative cases are shown. (**A**) Comparison between theoretical and experimental CRFs provides a good curve fit (blue and red lines are similar), but the total experimental time is long. (**B**) The fit performance is poor since theoretical and experimental curves are substantially different, but the total acquisition time of the experiment is short.

This study will address how to find the best fit of the CRF within the shortest period of neural recording. One possibility of improving the fit of the curve is increasing the number of contrast values experimentally, along with the number of trials and the duration of the visual stimuli (Figure 1A). However, adding more conditions increases the total recording time. Inversely, if one wishes to minimize the recording duration, the variation of neuronal responses to contrast will be considerably higher, and the CRF fit will be low (panel B). These two extremes are inadequate. Therefore, a point where a short recording time and an appropriate number of experimental conditions to yield a suitable CRF fit are required.

Here, we present a novel method to precisely and repeatedly obtain reliable CRF from big experimental data sets, offline or online, in a reasonable time. The method consisted to calculate a priori experimental variables that contribute the most to obtaining optimal CRF curves in a short period. To that end, from experiments with great contrast, many repetitions and a long trial duration, a “ground truth” CRF was formed. From this “theoretical” CRF, multiple data subsets were drawn to build representative new CRFs. An error between the ground truth CRF and the new CRFs was calculated, and this quantification was evaluated to validate that what formed CRF was the most similar to the theoretical ground truth. Thus, experimental CRFs were ranked by their adjustment to this “theoretical curve”. Such an adjustment was defined as the performance of the fit. In parallel, the experimental time of each CRF was estimated, and combined with the performance of the fit to obtain the optimal combination of parameters searched.

Since the number of iterations to generate this data set can be extremely large, we first simulated CRFs with a nonlinear-Poisson cascade model. The nonlinear function was the NRE, which was the theoretical ground under the given experimental conditions. Such experimental conditions were the number of trials, the amount of input contrast levels, the length of stimulus used, and different types of contrast metrics (e.g., linear or logarithmic scales). Then, “experimental” Poisson spiking data from the theoretical curve were generated, and from these average data points, new CRFs were fitted with the NRE. Thus, the performance of the fit was calculated by the comparison between theoretical and experimental curves.

We selected CRFs that minimized the experimental recording duration and maximized the fit. Our simulations showed that a point where CRFs maximize the fit’s performance with a considerable experimentation time of recording exists. Such data sets of contrast curves were then experimentally tested in the visual cortex of cats to confirm the variables that best satisfy both requirements, best fit in a short recording period. Our analysis revealed that 4 sets of parameters with less or equal to 6 experimental visual contrasts satisfied our premise of obtaining good CRFs’ performance in a short recording period.

## 2. Material and Methods

For all experimental and theoretical data, CRFs were built by fitting points to the Naka-Rushton Equation (NRE). The NRE describes the behavior of visual neurons to contrast changes [6,7], and is defined as follows:

(1) $$r\left(c\right)=R_{max}\frac{c^{n}}{c^{n}+C_{50}^{n}}+B$$

where *r*(*c*) is the output response at contrast *c.* Parameters of the NRE are *B*, the baseline response, *n*, the exponent of the curve (it is related to how rapidly the curve transitions around its inflection point), *R_max_*, the dynamic range of the curve, and C_50_, is the half-saturation contrast constant.

### 2.1. Curve Fitting

The fitting of the experimental data points to obtain the CRF from the NRE was achieved through a nonlinear least squares curve fitting (LSCF) algorithm. The LSCF used was from the *lsqcurvefit* function of MATLAB (Mathworks, Natick, MA, USA). The data point to obtain such curve fit was obtained from the average of the mean firing rate (the spike count per second) of trials of an experiment. For example, one experiment consists of the visual presentation of 6 different contrast percentages for 2 s each. Each trial was repeated for 10 times. Then, to calculate the mean firing rate, we average each point of the data set for the number of spikes for 2 s, divided this point by the ten repetition trials. This average iterated for each of the six contrast levels. Thus, for this particular experiment, 6 average contrast levels were used to calculate the curve fitting of the CRF and their parameters from the NRE. The algorithm used for fitting curve parameters to data points was trusting region reflective [8], with the maximum number of iterations set at 1000, and a termination tolerance on the function value and the contrast, *c*, of 10^−^^11^ [9]. Initial values for curve parameters were drawn from a homogeneous distribution between limits imposed below.

The parameters of NRE were bounded to simulate the response of neurons to visual contrast in biologically possible ranges. C_50_ was bounded as (0, 100)%. Boundaries of C_50_ higher than 100% contrast show a misleading *R_max_*, that is much higher than the experimental dynamic range. A quick set of simulations revealed that the most stable boundary for *R_max_* was (0, MAX + MAXERR), where MAX was the maximum firing response to contrast, and MAXERR was the maximum uncertainty (2 standard deviations) of MAX (Supplementary Table S1). The baseline *B* was also settled with that range. Another set of simulations showed that the optimal boundary for *n* was (0, 6) (Figure S1 and Table S2). Imposing an upper boundary on *n* greater than 6 makes neither biological nor statistical sense (Figure S1, panel D), which is in agreement with biological measurements [10,11]. To avoid a potential local minimum as a possible global minimum per each curve, fitting LSCF was iterated with random NRE parameters (N = 500). Parameters were drawn from a homogeneous distribution with boundaries as above described. The global minimum was then conserved.

### 2.2. Computational Model

**Figure 2:**
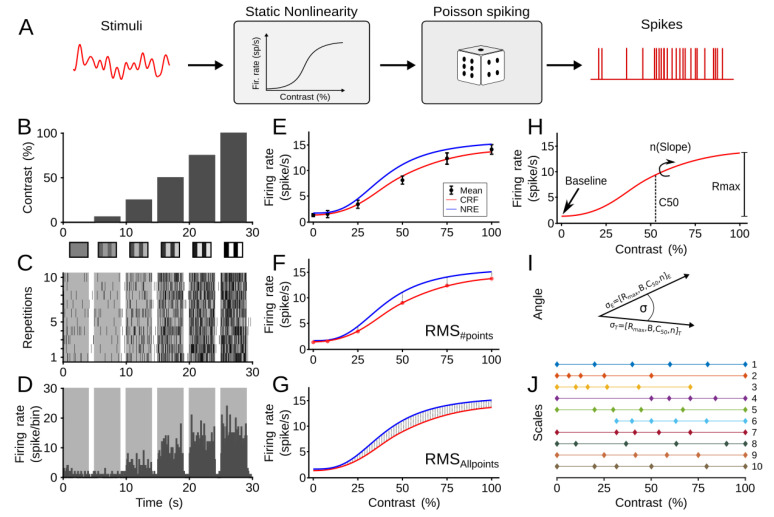
Simulating CRFs. (**A**) Model showing the spiking response to variation of visual contrast. Spikes were generated using an inhomogeneous Poisson process. For more explanations, see Section 2. The model is tested with different contrast magnitudes. (**B**) Visual contrast changing as a function of time. Visual contrast stimuli were presented randomly, but are shown aligned for better visualization. (**C**) Spike response to variations of contrast stimuli. (**D**) Peri-stimulus time histograms generated in order to confirm contrast responses. (**E**) CRF response based on the model output from the firing rate of the simulated neuron. The blue line indicates the theoretical curve with fixed contrast response parameters from the NKE. Black dots show average responses of spiking firing rate to a given contrast. The red line shows the CRF fit from the least-squares curve fitting. Testing conditions were preemptively determined and varied (stimulus trial length, number of trials, number of points). (**F**) Calculation of RMS_#points_ by comparing particular CRF points to the theoretical curve (red dots). (**G**) Calculation of RMS_Allpoints_ by comparing all experimental CRF points to the theoretical curve. (**H**) Calculation of experimental parameters from the CRF fit (*R_max_*, *B*, C_50_, and *n*). (**I**) Calculation of the angle formed by the difference between theoretical and experimental parameters’ vectors. (**J**) Ten scales (metrics) with different contrast distributions are shown. Only scales of 6 points are represented.

A simplified and modified linear-nonlinear-Poisson cascade model was used to simulate neuronal responses to variations of visual contrast percentages (Figure 2A) [12]. For simplicity, the linear component that represents RFs of cells was not considered. First, the visual stimulus was passed through a nonlinear function, which in our case is the NRE. For each of these simulations (i.e., visual experiment), the NRE was set with fixed parameters. Once the instantaneous firing rate of the neuron was recovered, it was later transformed into spike trains by an inhomogeneous Poisson process. As a visual input with different levels of contrast is presented randomly (Figure 2B), spikes were collected and averages of mean firing rates were calculated (Figure 2C,D). In summary, our model generated dynamic spike responses from visual contrast inputs by using the NRE.

This model was used to obtain theoretical and “experimental” CRFs. While the ground truth CRF of the model was the NRE, experimental curves were obtained by simulating the model with different conditions (i.e., #points, #trials, trial length) (Figure 2E). At these particular experimental conditions, curve fitting was calculated and an experimental CRF was recovered (see Curve Fitting). Finally, the theoretical CRF was compared to the “experimental” CRF, and such a difference was quantified as a measure of the error of the experimental conditions used. In our case, it should be clarified that simulated spike trains are equivalent to single-unit but not multi-unit recordings.

### 2.3. Optimization Criteria

Our criteria is to find the best curve fit in a short recording period that a neuron has. To optimize such a premise, we quantified the performance of the fit and the total duration of the experiment. As Figure 1 shows, it is expected that the performance of the fit increases when the duration of the experiment is long. Inversely, the performance of the fit is poor when the experiment duration is too short. Between these two extremes, a neuron has a point where the fit performance equals the total experiment duration. Therefore, the performance of a neuron is maximized where the curves that describe the fit performance and the total experiment duration overlap.

#### 2.3.1. Error Estimation of CRF

As explained above, theoretical and experimental CRFs were recovered, and an estimate from their comparison is needed (Figure 2E). The curve fitting recovers two outputs, the CRF curve and the parameters of the NRE. For the theoretical ground truth, these variables were known a priori. To evaluate differences between theoretical and experimental outputs, two methods were used. To evaluate the differences between theoretical and experimental outputs, we used three methods. Outcomes of these three methods will be compared to search for consistent experimental conditions among repetitions, and thus, find a regularity in the analysis.

The first approach consisted of the direct comparison of the two CRF curves, the theoretical and experimental curves. The second method quantifies the difference between theoretical and experimental parameters of the NRE.

For the first method, we used the root-mean-square (RMS) error of the CRF. RMS is defined as:

(2) $$RMS=\sqrt{\frac{\sum_{i=1}^{N}{\left(E_{i}-T_{i}\right)}^{2}}{N}}$$

where *N* is the total number of *i* points on the CRF, *E*_*i*_ is the experimental data, and *T*_*i*_ is the theoretical curve. In parallel, RMS was used with all the points of the CRF curve (*N* = 100). Error estimation by the number of points or all points were defined as RMS_#points_ (Figure 2F) or RMS_allpoints_ (Figure 2G), respectively. These two measurements were calculated to compare the differences between a discrete and continuous use of fitting of errors.

Differences between theoretical and experimental parameters of the NRE were compared by calculating the angle difference between two vectors (Figure 2H). These vectors were formed by experimental or theoretical parameters, so $\sigma_{E}={\left[R_{max},B,C_{50},n\right]}_{E}$, or $\sigma_{T}={\left[R_{max},B,C_{50},n\right]}_{T}$, respectively. The angle difference is calculated by the dot product of the two vectors, divided by the length of the two vectors, so:

(3) $$\sigma =arccos\left(\frac{\sigma_{E}\cdot \sigma_{T}}{\left|\sigma_{E}\right|\left|\sigma_{T}\right|}\right)$$

where $\left|\sigma_{A}\right|=\sqrt{R_{max}^{2}+B^{2}+C_{50}^{2}+n^{2}}$. If vectors are similar, and so are the parameters, the angle becomes small, otherwise the angle is large.

#### 2.3.2. Temporal Error Estimation

Total experimental time was calculated by multiplying experimental conditions. For example, for the previous example of 6 visual contrast levels with 2 s duration, repeated 10 times, the total time was 6 × 2 × 10 = 120 s. This factor was used logarithmically to enhance its importance.

#### 2.3.3. Monte Carlo Method

Monte Carlo simulations were used here to recover experimental CRFs and parameters of the NRE. These outputs were compared with the theoretical curves whose curves and parameters are known. Seven CRF parameter variations were considered: four of which are functional parameters (*R*_*max*_, C_50_, *n*, *B*), and three of which were experimental conditions (#points, #trials, trial length) (Table 1). A total of 10,000 replicates were used per experimental condition. On a side note, since there were four functional parameters (degrees of freedom), the absolute minimal number of data points for a fit to occur must be at least 4.

### 2.4. Metric Spacing or Scales

Different percentage distances between visual contrasts can be used to fit the CRF. Parametric and logarithmic scales are the most used scale in the literature (Figure 2J, scales 1 and 2, respectively). A logarithmic scale generates *n* spaced points between decades 10*^a^* and 10*^b^*, where *a* and *b* are the lower and upper bounds, respectively. The resulting vector, for both linear and logarithmic scales, was weighted by 100 to obtain percentages of contrasts. Since the duration of the experiment is unaffected by the different spacing of contrast, we investigated whether the position of the contrasts and their type of distribution influenced the performance of the fit. Table 2 details such distance and distribution of the ten scales used. While scales 2, 3, 4, 5, 6, 7, and 10 have a logarithmic distribution, scales 1, 8, and 9 have a linear distribution. Scale 3 lacks the last contrast at 100%. Scale 4 has contrasts concentrated around 100% (second half of the distribution). Scale 5 has contrasts concentrated around 25%. Scale 6 is similar to scale 5 but without the first contrast (0%). Scale 7 has contrast concentrated around 50%. Scale 8 is similar to scale 7 but linearly spaced, so visual contrasts have a tendency to be close to borders (0% and 100%). Scale 9 is similar to scale 8, but contrasts are less distributed towards the borders. Scale 10 has values logarithmically concentrated around 50%. These scales and their metrics were analyzed theoretically and experimentally.

### 2.5. Experimental Conditions

#### 2.5.1. Animal Preparation

Recording experiments were carried out on healthy adult female cats weighing between 3 and 4 kg. A total number of 2 cats (n = 2) were used in the current study. Using mathematical simulations, the use of animals required to validate theoretical results was reduced to a minimum (See Section 2.2). All surgical and experimental procedures were done according to the guidelines of the Canadian Council on Animal Care and were approved by the Ethics Committee of University of Montreal (CDEA 19-008), which ensures and complies with the commonly accepted “3R’s” (replacement of animals by alternatives wherever possible; reduction in the number of animals used, and refinement of experimental conditions and procedures to minimize the harm to animals). Prior to surgery, cats received subcutaneously a solution of atropine (0.1 mg/kg) and acepromazine (Atravet ^®^ 1 mg/kg, Boehringer Ingelheim Canada, Burlington, ON, Canada), to reduce the parasympathetic effects of isofluorane anesthesia and to induce sedation, respectively, and then they were acclimatized to the laboratory to reduce any stress. Anesthesia was induced with 3.5% isofluorane in a 50:50 (vol/vol) gas mixture of O_2_ and N_2_O. A catheter was placed in the cephalic vein to provide intravenous access. A tracheotomy was performed prior to the transfer of the animal to the stereotaxic apparatus. Following anesthetic induction, isofluorane concentration was maintained at 1.5% during surgical procedures. During recording sessions, the anesthesia was changed to Halothane (0.5–0.8%) in a 30:70 (vol/vol) gas mixture of O_2_ and N_2_O. The anesthesia level was continuously monitored in case it needed to be adjusted to reduce any animal stress and/or harm situation. Lubricant eye gel (Systane Gel drops ^®^, ALCON Fort Worth, TX, USA) was applied to avoid corneal dehydration. Oxygen saturation was monitored using a pulse oximeter, cardiac activity was monitored throughout the experiment by recording the ECG and the animal’s temperature was maintained at 37 °C by means of a heated blanket controlled by a rectal thermometer probe. A bolus intravenous injection of 2% gallamine triethiodide was administered to induce muscular paralysis and, subsequently, the animal was placed under artificial ventilation. A 1:1 (vol/vol) solution of 2% gallamine triethiodide (10 mg/kg/h) in 5% of dextrose in lactated ringer was continuously administered intravenously to maintain muscular relaxation and to provide nutrition and electrolytes. Expired levels of CO_2_ were maintained between 35 and 40 mmHg by adjusting the tidal volume and respiratory rate. The animal’s heart rate was maintained at 180 bpm ± 10. Pupils were dilated using atropine (Mydriacyl ^®^, ALCON, Fort Worth, TX, USA) and nictitating membranes were retracted using phenylephrine (Midfrin ^®^). Rigid contact lenses of appropriate power were applied to the corneas. The lubricant eye gel was replaced by a liquid one (Blink^®^, ABBOTT, JOHNSON & JOHNSON, Irvine, Californie, États-Unis) during recordings and was used when needed. Craniotomies were performed to provide access to areas 17 (4–8 P; 0.5–2 L, Horsley-Clarke coordinates), 18 (1–4 A, 2–7 L) and 21a (2–6 P; 7–11 L).

#### 2.5.2. Visual Stimuli

Visual stimuli were generated using the VPixx software (Vpixx Technologies Inc., St-Bruno, QC, Canada) and images were projected onto an isoluminant screen at a viewing distance of 57 cm, with a refreshing rate of 60 Hz. The images covered 116° by 150° of visual angle, with a mean luminance of 50 cd/m^2^. To avoid modulation surround effects of different size stimuli when the visual contrast is varied [13], we stimulated receptive fields monocularly with full field drifting sinusoidal gratings presented at various directions from 0° to 330° with 30° steps. Extracellular recordings were carried out through all layers of the visual cortex. Responses in areas 17, 18 and 21a, and in the posteromedial lateral suprasylvian cortex (PMLS) were recorded simultaneously. The spatial and temporal frequencies used were within the stimulation range of areas 17 and 21a (SF = 0.3 cycle/deg and TF = 3 Hz), and of area 18 and PMLS cortex (SF = 0.2 cycle/deg and TF = 4 Hz) [14,15,16,17] In total, 24 contrast values (0, 3, 6, 8.5, 12, 17, 21, 26, 29, 32, 35, 38, 41, 44, 48, 53, 57, 63.5, 70, 74.5, 79, 83, 91, and 100 %) were tested. The presentation of the combinations of directions and contrasts was randomized to avoid hysteresis that can shape CRF (i.e., cat striate neurons show hysteresis) [18]. Each stimulus was presented 50 times and lasted 4 s. The neural response obtained a 0% contrast was used as a blank.

#### 2.5.3. Electrophysiological Recordings and Data Acquisition

Extracellular activity was recorded using linear multielectrodes of 32 channels (impedance at recording sites were between 1 and 2 MOhm, A1x32-6mm-50-177, Neuronexus). Efforts were made to record cortical neurons with the most centrally located receptive fields, using the visuotopic maps of the targeted visual areas. Electrodes were lowered at 3 mm depth perpendicular to the surface. Electrophysiological signals were acquired at 30 KHz and band-pass filtered 1–7500 Hz using an open-source system (Open-Ephys platform) [19]. Single-unit clusters were identified using the software package Klusta [20]. Manual validation verified the selected clusters. The firing rate of neurons was quantified as the spike count over a time window duration. Units with very low firing rates at maximum contrast magnitude (<3 spikes/s) were excluded from the analysis. Data was analyzed with custom scripts in MATLAB (Mathworks, Natick, MA, USA). A total of 42 neurons were used for the theoretical validation.

#### 2.5.4. Experiment Termination

At the end of the experiment, animals were euthanized by an intravenous injection of sodium pentobarbital (Euthanyl, 110 mg/kg, Bimeda -MTC Animal Health, Cambridge, ON, Canada). Animals were transcardially perfused with a phosphate-buffered solution (PBS 0.1M, pH 7.4) followed by a fixative (Paraformaldehyde 4%, Fisher Scientifc, Ottawa, ON, Canada). Brain tissue was cryoprotected using sucrose solutions at different concentrations (10 to 30%), frozen and stored at −80 °C. Then, 40 µm coronal sections were obtained and subsequently stained and used to reconstruct the electrodes’ position.

### 2.6. Statistical Analysis and Data Analysis

No animals were excluded from the analysis. All data were also analyzed using Matlab functions (MATLAB 2018; Math Works Inc., Natick, MA, USA). Data extraction and analysis were performed blindly. The parametric test ANOVA was used to compare differences between groups. When the test revealed significant differences, multiple comparisons were performed using Tukey’s tests, and *p* values were revealed. A bootstrap method was used to test significance for experimental conditions and the recording time they generated. This analysis iterated experimental conditions (i.e., #points, #trials, trial length) to calculate the theoretical patterns’ average error.

## 3. Results

The results are divided into two sections. In the first section, theoretical results show what are the experimental conditions (i.e., #points, #trials, trial length) that minimizes the recording time and maximize the fit performance. Such a set of conditions is tested experimentally in the second section.

### 3.1. Theoretical Results

To search for experimental conditions that minimizes the recording time and maximizes the fit performance, we used a simplified cascade nonlinear-Poisson spiking model that reproduces theoretical and experimental curves. While theoretical curves were obtained by simulating NRE, experimental ones were obtained by fitting simulated neuronal mean firing rates to stimuli of varied contrasts (Figure 1). Comparison between the theoretical and experimental curves measured the fit performance (Equation (2)). The recording time was calculated by the multiplication of experimental conditions. Thus, this model was used to search curve fits with the shortest experimental time and the best fitting performance.

**Figure 3:**
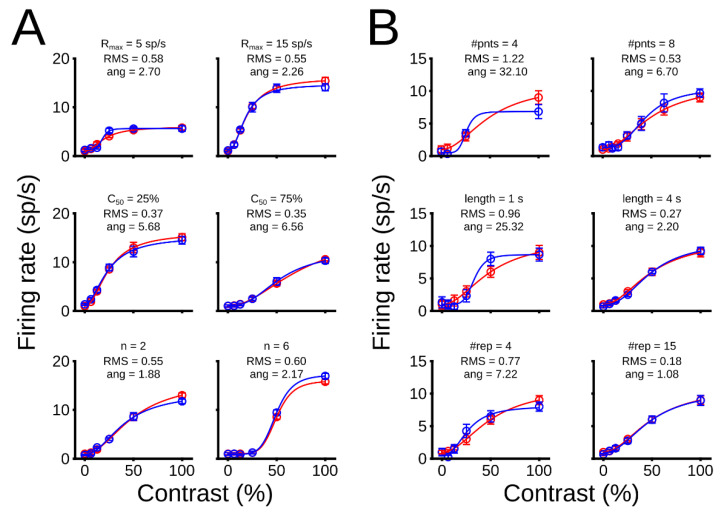
Examples of simulations of CRFs for different parameters and experimental conditions. (**A**) Variation of parameters of CRFs. Top, middle and bottom panels show changes of *R_max_*, C_50_, and *n*, respectively. Parameters for simulations in top panels with C_50_ = 20%, *n* = 2, and B = 1 sp/s/; middle panels *R_max_* = 15 sp/s, *n* = 2, and B = 1 sp/s; bottom panels, *R_max_* = 15, C_50_ = 25%, and B = 1 sp/s. For this set of simulations, number of points = 6, trial length = 2 s, and number of repetitions = 10. (**B**) Variation of conditions of CRFs. Top, middle and bottom panels show changes of the number of points (#pnts), length of trials (len), and number of repetitions (#rep), respectively. Conditions for simulations in top panels, len = 1, and #rep = 6; middle panels, #pnts = 6, and #reps = 6; bottom panels, #pnts = 6, len = 1. For this set of simulations, *R_max_* = 10, C_50_ = 50%, B = 1 sp/s, and *n* = 2. A logarithmic scale was used for all these simulations.

*Examples of simulations of CRFs.* We simulated nine representative sets of parameters and conditions to visualize how the model accomplishes our premise. Here, we only analyzed how the performance of the fit is affected. As Figure 3 shows, theoretical (red line) and experimental (blue line) curves are depicted. Experimental curves are calculated from the fit of data points (black dots). For simplicity, only the logarithmic scale is shown in these examples. First, NRE’s parameters were varied, and conditions remained fixed (Panel A), then experimental conditions were changed, and parameters were preserved (Panel B).

In general, the variation of parameters affects the performance of the fit slightly. In Figure 3A top panels, the saturation of the CRF, the *R_max_*, was increased from low to high levels. The RMS_#points_ changed 2.53%, from *R_max_* = 5 sp/s to *R_max_* = 15 sp/sec. Similar results were observed when the position of the CRF, C_50_, shifted from left (C_50_ = 25 %) to right (C_50_ = 75 %), showing a small RMS_#points_ variation of 2.07% (middle panels). Increasing the curve’s steepness, *n*, affected the RMS_#points_ by 4.71% (bottom panels) from *n* = 2 to *n* = 6. The baseline, B, was not analyzed to simplify the description. This latter parameter affected the performance of the fit when its value was larger than half of maximum firing rate, meaning that the limit for a good fit was *R_max_*/2 = B.

Contrary to the variation of parameters, variation of conditions largely affected the CRF’s fit. Results of these simulations can be seen in In Figure 3B. The addition of data points enhanced the performance (minimized the RMS_#points_) of the fit by 38.22% from 4 to 8 points. Increasing the length of the trial also allowed a better performance, in which the RMS_#points_ decreased 55.78% from 1 s to 4 s duration. Finally, the number of repetitions showed the largest fit improvement. The RMS_#points_ decreased from 62.05%, when repetitions moved from 4 to 15. Taken together, variation of parameters improved little the performance of the fit, whereas changing experimental conditions modified the CRF’s fit substantially.

#### 3.1.1. Theoretical Optimization of Experimental Conditions

As shown above, the performance of the fit depended more on the experimental conditions than on the CRF parameters used. To obtain a robust sight of how the performance of the fit is affected by the experimental conditions, we averaged CRFs simulated overall parameters quantified. To that end, Monte-Carlo methods were used by repeating several CRFs with a random sampling of NRE parameters, and the mean error for the three estimators (RMS_#points_, RMS_Allpnts_, and angular difference) was calculated. Thus, the profile of each experimental condition was obtained from averaging CRF simulations of varied NRE parameters.

**Figure 4:**
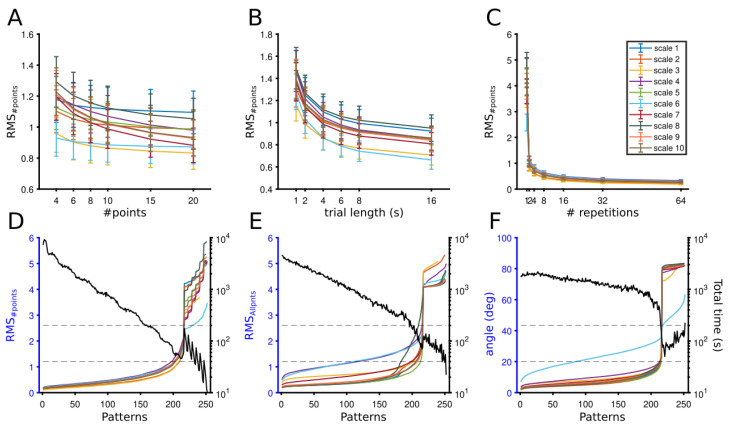
Optimization of fit performance and total experimental time to experimental conditions across metrics (errors). Fit performance as a function of experimental conditions is shown in (**A**–**C**). Each dot is the average of 300 CRF simulations. Bars represent SEMs. (**A**) RMS_#points_ for a number of points. (**B**) RMS_#points_ for the length of trials. (**C**) RMS_#points_ for a number of repetitions. Sorting of RMS_#points_ (**D**), RMS_Allpoints_ (**E**), and angles (**F**) solutions for combination of all patterns, experimental conditions (left y-axis), against the average total experimental time (black line, right y-axis). Lines are the average of 174 CRF simulations. Dash lines are the range of optimal fit performance and experimental duration.

The quantification of such mean error is described in Figure 4. Panels A, B and C show the performance of the fit as a function of the number of points, the trial length, and the number of repetitions, respectively. For each experimental condition, all the 10-metric scales were considered. Each point of the curves was the average of 174 different CRF simulations, and error bar represented the SEM. While the performance of the fit was quantified with RMS_#points_, lower levels of this estimator reflect a higher performance of the fit.

*Experimental conditions and performance of the fit.* In general, the performance of the fit increased (RMS_#points_ decreased) when experimental conditions became large. For example, as the number of points to fit the CRF increased, the RMS_#points_ decreased slightly. In this case, only scale 7 had significant differences between RMS_#points_ values when the number of points increased (*p*-value = 0.025, One-way ANOVA). A similar tendency was observed for the performance of the fit when the length of trials was large. The RMS_# points_ for all scales dropped when the trial length increased (all scales, *p*-values < 0.001, One-way ANOVA). Beyond 4 s duration, RMS_#points_ differences between the length of trials and scales tended to be constant. This decline of RMS_#points_ was even more drastic for the number of repetitions. All scales showed significant differences as the number of trials increased (all scales, *p*-values < 1 × 10^−8^, One-way ANOVA). Beyond four repetitions, the performance of the fit was constant, and between 8 and 64 repetitions, no significant differences were revealed for all scales (*p* < 0.05). Means (±sem) of the last RMS_#points_ value for the number of points, trial length, and the number of repetitions over all scales were 0.955 (±0.03), 0.832 (±0.023), and 0.269 (±0.012), respectively with significant difference between them (*p*-values = 3.5 × 10^−18^, One-way ANOVA). Thus, increasing the number of repetitions, then the length of trials, and to a lesser degree, the number of points improved the performance of the fit.

#### 3.1.2. Optimization Point

Although the performance of the fit improves as the size of the experimental conditions increases, this improvement comes with a trade-off. The recording time also increases. To assure optimal performances of the fit with considerable recording times, we searched for a point where the two constraints were equal. An optimal point of a neuron (CRF optimization) was defined where the curves that describe fit performance and total experiment duration overlap. This optimization was searched as follows.

To find such neuron’s CRF optimization, we combined the three experimental conditions with the generated experimental time. Each experimental combination was defined as a “pattern”, and expressed as a tuple of values {#points, #trials, trial length}. The fit performance and the experimental time were calculated from the 252 patterns used (6 #points, 7 #trials, 6 trial lengths). The recording time was calculated by averaging the recording time for each pattern of the 10 scales used.

We defined an optimal point when the recording time and the fit performance were equal. Since the recording time decreased as the error estimator’s curves increased, the optimization CRF point was the interception between these two curves. Such results are depicted in Figure 4D–F, in which the RMS_#points_, RMS_Allpnts_, and angular differences are shown, respectively. For the 252 patterns, curves representing the fit performance of the ten scales are in the left y-axis, and the curve formed by the recording time is in the right y-axis (black line). For simplicity, only averages are shown as each curve resulted from 174 randomly simulated parameters. A logarithm scale for the recording time was used to highlight the importance of this factor.

*Maximization to find the optimal point*. An optimal point was obtained between error estimators and recording time for the ten different scales used. For the three error estimators, optimal points were located between 200 to 225 patterns and around 100 s (between 30–200 s) of the recording time. Slight differences appeared in the profile of each estimator, in particular for the scale 6. For RMS_#points_, when only the number of dots was considered to form the fit, all curves increased suddenly, but scale 6 deviated after the intersection with the temporal recording curve (Figure 4D). These fluctuations rose as the fit’s error estimator was computed by considering the total number of dots of the CRF (N = 100). Such an estimator is the RMS_Allpnts_. Scales 6 and 7 had lower performance, whereas for scale 8 the fit performance increased a bit early than the other scales at the same patterns (Figure 4E). Scale 6 deviated further for the angle differences that quantified the similarity of CRF parameters between the theoretical and experimental curves (see Section 2). Here, smaller angles meant a better-fit performance of patterns. The angle error raised almost equally for the same range of patterns for the other scales, and the intersection with the recording time was almost at the same point (Figure 4F). Taken together, we found optimization points from theoretical simulations that examined which experimental conditions improved the performance of the fit for CRF quantification. In order to verify such results, we tested these optimized points on experimental data obtained from the cat’s visual cortex.

### 3.2. Experimental Results

Theoretical values that minimized both the error and the experimental time were tested empirically. These points correspond to the intersection between the performance of the fit and the total experimental time calculated as above. Each of these intersections gave a particular combination of three elements or “patterns”: number of contrast points, number of trials, and duration of each trial in seconds. Patterns were also examined for the ten contrast scales. These combinations enabled us to test the desired patterns that maximized the time versus fit performance.

**Figure 5:**
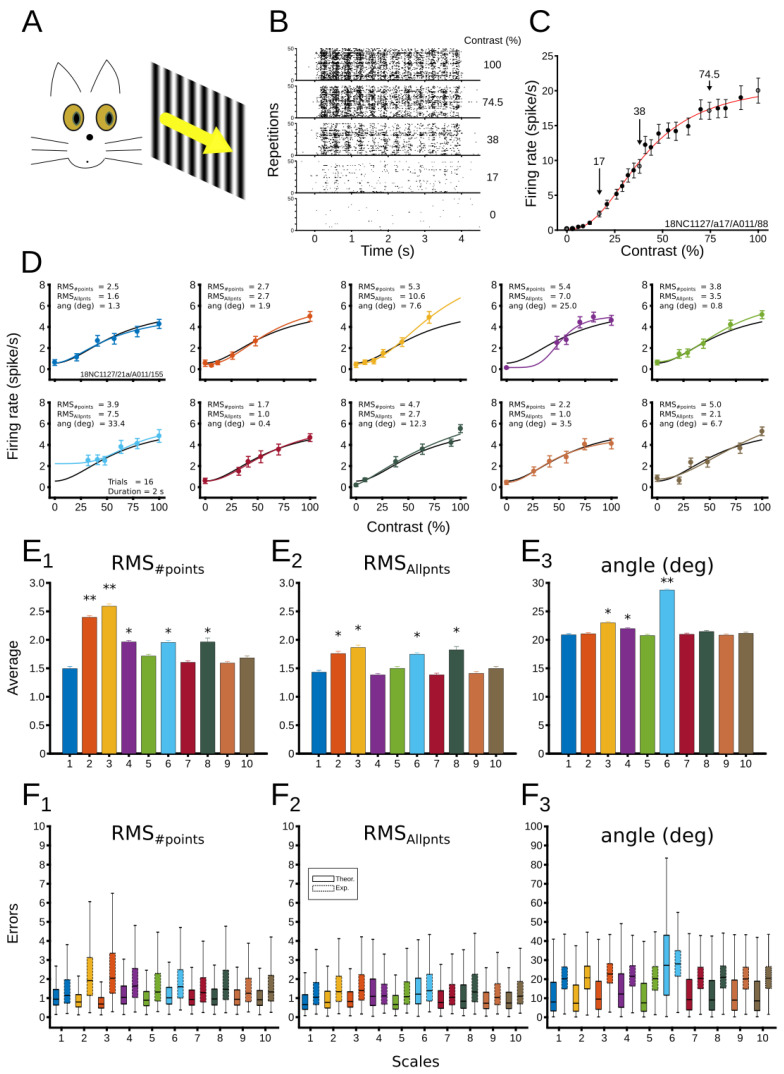
Experimental validation of computational simulations. (**A**) Experimental setup. Visual stimuli presented to cortical neurons in anesthetized cats. Contrast stimuli were presented randomly. (**B**) Representative raster plots of cortical neural responses. (**C**) CRF of recorded neurons, considered as the ground truth curve. Arrows indicate contrast used in (**B**). (**D**) Representative comparison between the empirical CRF (ground truth, black line) and the ten tested scales (color lines from scale 1 to 10). Errors are indicated in each subplot. Number of trials, number of points and trial length were similar across CRFs. (**E1**–**E3**) Average errors of scales across all patterns from 42 neurons. Best scales are those minimizing the error. Significant differences between scales are expressed as * *p* < 0.05; ** *p* < 0.01. Significant differences for each average error, (**1**) RMS_#points_, *p* = 1.0 × 10^−97^; (**2**) RMS_Allpnts_, *p* = 2.9 × 10^−35^; (**3**) Angle, *p* = 2.6 × 10^−245^; One-way ANOVA. (**F1**–**F3**) Boxplots between simulated (solid line) and empirical (dashed line) data for the 3 different errors across the scales. Lines inside boxplots show medians, and whiskers indicate 95% confidence intervals.

Extracellular recordings of visual neurons (n = 42) of the cat’s cortical areas 17, 18, 21a and PMLS were used to test these patterns experimentally. The contrast sensitivity was quantified by presenting drifting sinusoidal gratings at the preferred direction of neurons (Figure 5A). To ensure that we covered the broadest range of patterns as large as possible (81 patterns), we tested 24 different contrasts values, each repeated 50 times for 4 s (Figure 5B). The experiment lasted approximately 2 h. The 24 contrast points were chosen so that all types of scales could be examined. A ground truth CRF for each cell was calculated from these 24 contrasts (Figure 5C), and, similar to the theoretical section, it was defined as the “theoretical” CRF (Figure 5C,D Left). Forty-two theoretical CRFs were formed from the combination of optimized experimental conditions.

#### 3.2.1. Evaluation of Patterns’ Performance

Such CRFs were used afterwards as a template for calculating the optimization of each pattern. From the theoretical CRF, subsets of CRFs were formed by the experimental conditions characterized in a pattern. For example, for a pattern with {6, 16, 2.0} conditions, 6 of the 24 percentage of contrasts, 12 of 50 repetitions, and the first two seconds of the stimulus time recording were selected as experimental conditions (Figure 5D). This sample was iterated randomly (100 iterations) from the ground truth data. Each pattern performance was quantified for each scale. Thus, the performance of each pattern was evaluated by comparing subsets of “experimental” data against the ground truth data.

The performance of scales for all neurons was first analyzed with the average output of RMS_#points_, RMS_Allpnts_, and the angle between vectors. Results are shown in Figure 5E, in which significant differences between scales were found. For RMS_#points_, scales 2, 3, 4, 6 and 8 showed lower performance than the rest of the scales (Figure 5E1). Scale 1 had the best performance. Similar effects were observed for the RMS_Allpnts_, but average differences between scales were less distant (Figure 5E). For instance, scales 2 and 3 showed less significant differences with respect to other scales than in RMS_#points_. Scale 7 had the best performance, and scale 4 had the poorest performance. In the average angle, it is scale 6 that had a large difference from the other scales (Figure 5E3). In summary, depending on the type of error used, scales 2, 3, 4, and 6 showed a poor performance (For more details of the comparison see Supplementary Table S3). In general, the simulated data tend to cover the empirical data when comparing the distribution of the recorded experimental conditions with the theoretical simulations for each scale (the 24 visual contrasts to test the 81 experimental conditions) (Figure 5F).

**Figure 6:**
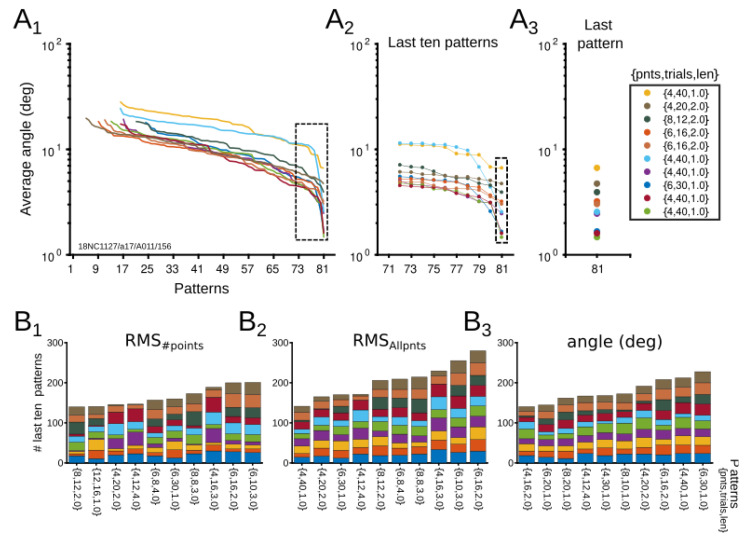
Experimental patterns with the best performance. (**A1**–**A3**) Representative example for angle errors sorted from the worst to the best pattern performance. Best patterns are those minimizing the error. (**A1**) Errors across all patterns, (**A2**) Errors of the last ten patterns, (**A3**) Last pattern that minimizes most of the error. (**B1**–**B3**) Histogram showing the most representative patterns that minimize the error for all spacing scales. Data was taken from last ten patterns (**A2**). Last ten pattern errors for RMS_#points_ (**B1**), RMS_Allpnts_ (**B2**), and angle difference between vectors (**B3**).

*Most representative patterns.* Now, in order to find patterns that were representative, we searched patterns that minimized average errors (maximized the fit performance). For every tested neuron, error performance was ranked from the largest to the smallest value. From this sorting, the last ten patterns that minimized most average errors were saved. Each scale was also measured. Figure 6A shows a representative example of this procedure. Here, RMS_#points_ was quantified for each scale, and after sorting the performance of each sequence, the ten patterns that minimized most average errors were analyzed (Figure 6A2). The results of the last ten patterns for each neuron were also collected for RMS_Allpnts_ and angle differences between vectors.

Patterns were compared to identify representative experimental conditions that maximize the fit performance. Figure 6B shows stacked bars of the ten more represented patterns for the different scales. Across errors, different patterns emerged as the most recurrent. Those patterns that appear in the three types of errors and ranked from the most to the less repeated were: {6, 16, 2.0}, {6, 30, 1.0}, {4, 12, 4.0}, and {4, 20, 2.0}. Ranking and appearing in two errors, the patterns were {6, 10, 3.0}, {4, 16, 3.0}, {8, 8, 3.0}, {6, 8, 4.0}, {4, 40, 1}, and {8, 12, 2}. The recording time of such patterns ranged between 160 and 192 s.

**Figure 7:**
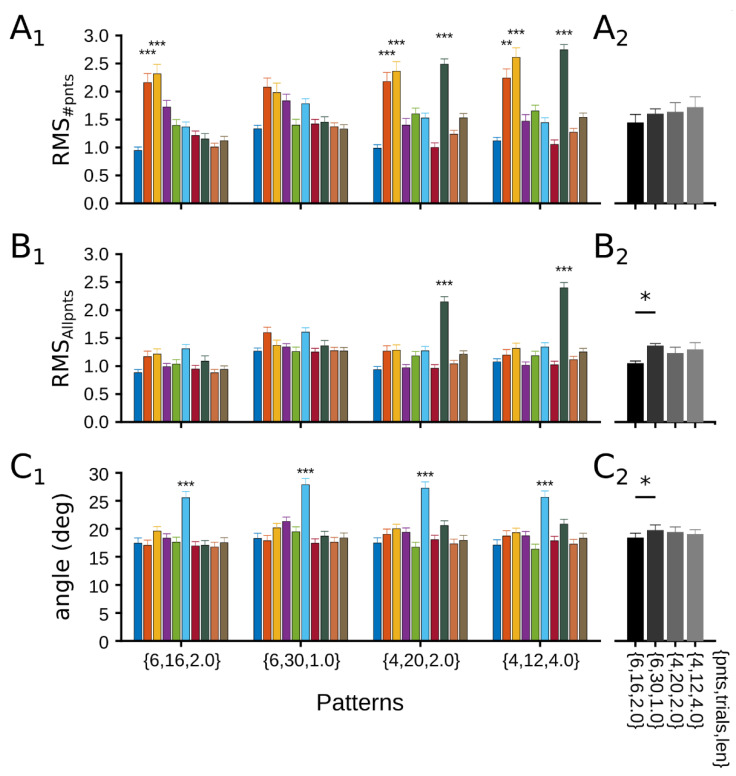
Performance of the four best patterns. (**1**) Average errors of patterns across scales. (**2**) Mean errors for each pattern as scale outputs are averaged in a single value. Bars show the average errors for RMS_#points_ (**A**), RMS_Allpnts_ (**B**), and angle difference between vectors (**C**) across scales (**1**) or mean errors (**2**). * *p* < 0.05; ** *p* < 0.01; *** *p* < 0.001. Statistical comparisons for figures in 1 are not shown. For more details of the statistical comparisons refers to Supplementary Tables S4 and S5.

The four most significant patterns were further investigated by averaging their errors across all the neurons analyzed. As Figure 7 shows, their error profiles were similar to those of the average error of all the patterns (Figure 5E). For example, scale 8 had a high error of RMS_#points_ and RMS_Allpnts_ for patterns {4, 12, 4.0}, and {4, 20, 2.0}, scales 2 and 3 had a large RMS_#points_ for the four patterns, and scale 6 had a greater increase of the vector angle for the four patterns. Another remark of this quantification was that the performance of a pattern depended strongly on the scale and error analyzed. For instance, for pattern {6, 16, 2.0} scale 2, RMS_#points_ was large but small for the angle vector. The performance of each pattern was summarized by averaging errors across all scales (Figure 7A2–C2). Although for RMS_#points_, pattern {6, 16, 2.0} has the smallest mean error (1.44 ± 0.47), so with the best performance, no differences were found between patterns (*p*-values = 0.222, Two-way ANOVA). For RMS_Allpnts_ and angle vector errors, patterns’ performance was similar to the average error of all patterns, and for the two comparisons, the pattern {6, 16, 2.0} had a smaller average error than the pattern {6, 30, 1.0} (*p*-value < 0.01). Taken together, four patterns that minimize errors emerged as the most recurrent, and the maximization of their fit performances depended on the scale and error quantified.

**Figure 8:**
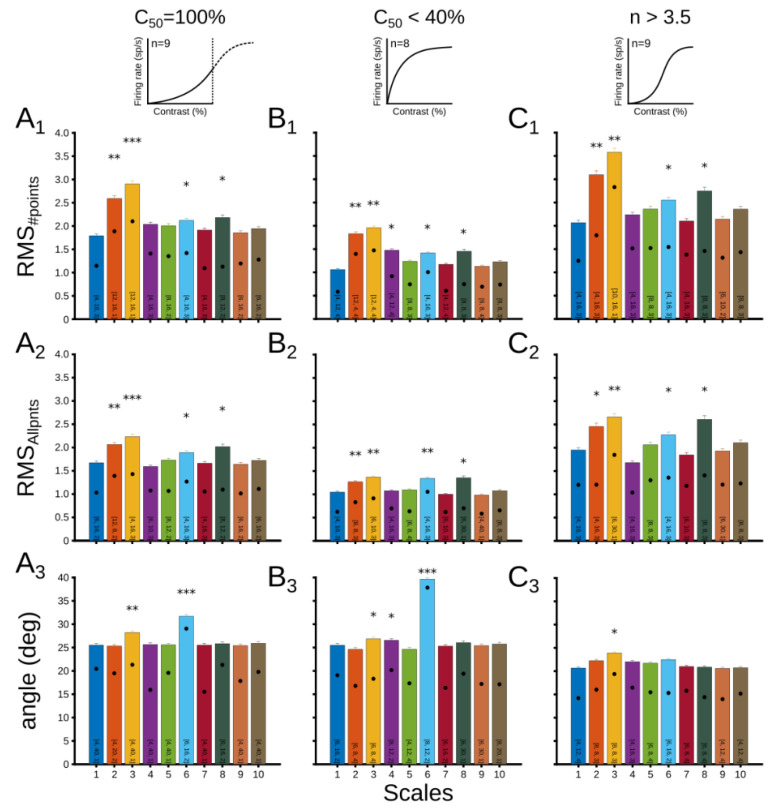
Particular cases of CRF across scales. (**A**) CRFs with C_50_ = 100%, (**B**) CRFs wih C_50_ < 40%, (**C**) CRFs with n > 3.5. Average errors for (**1**) RMS_#points_, (**2**) RMS_Allpnts_, and (**3**) angle difference between vectors. Each bar shows the pattern that minimizes most errors, and the black dot shows the position in the average range. * *p* < 0.05; ** *p* < 0.01; *** *p* < 0.001.

*Particular cases of CRF.* We tested if different points of the curve were more informative than others and may require different amounts of sampling. For example, points on the curve where they begin to accelerate or flatten again after the maximum steep are particularly useful. So, to explore how experimental conditions allocate across these points, we studied three extreme cases: (i) C_50_ = 100%, (ii) C_50_ < 40%; (iii) n > 3.5 (Figure 8). In the first case, when C_50_ is saturated, the factor tends to be low, showing almost linear CRFs (Supplementary Figure S2). In case 2, when C_50_ is low, the curves tend to have a rapidly saturating hyperbolic-like profile. Only 3 CRFs presented this profile when C_50_ < 30%. Unlike case 1, in case 3, the curves present a steep slope.

Figure 8 shows the result of this analysis, where the three errors were measured as a function of three particular cases for each scale. Each scale’s bar also describes the pattern that achieved a minimal error. When C_50_ = 100%, while RMS_#points_, and RMS_Allpnts_ showed similar errors to Figure 5E, in which scales 2 and 3 had the lowest performance, the angle error showed scales 3 and 6 as the worst. As C_50_ increased (Panel C), RMS_#points_ and RMSAllpnts maintained profiles, but average errors were less pronounced. For the angle, scale 6 stood out as the worst, and the best were scales 2 and 5. Errors were higher for the third case when n was large (Panel C). Besides scales 2 and 3, scale 8 also had a low performance for RMS_#points_ and RMS_Allpnts_. For the angle, only scale 3 had a high error.

For the angle error, patterns minimizing the error showed a tendency across cases. When C_50_ saturated, these patterns had more repetitions, fewer contrast points to the sample, and short periods of recordings. On the contrary, as n increased, the number of sample points increased, the number of repetitions decreased, and the recording time was longer. When C_50_ < 40%, linear scales were likely to have more repetitions, short recording times, and an intermediate number of contrast points, compared to exponential scales that were characterized by a greater sampling of contrasts, longer times, and fewer repetitions. This result shows that curves that tend to be more linear optimize for more repetitions, while nonlinear CRFs are more likely to increase the number of contrast sampling.

#### 3.2.2. Dynamical CRF Characterization

One caveat of the theoretical approach is that the spiking model lacked neuronal adaptation. Since the visual contrast is constant over time, a gradual decrease of neuronal responses is produced long the stimulation. We investigate whether short or long-duration trials can influence the fit of CRF. In turn, this analysis may allow choosing what pattern is the most representative to obtain a good fit performance in a short period. Furthermore, the dynamic response of CRFs to long periods, several contrast points, and several trials has not been thoroughly studied.

**Figure 9:**
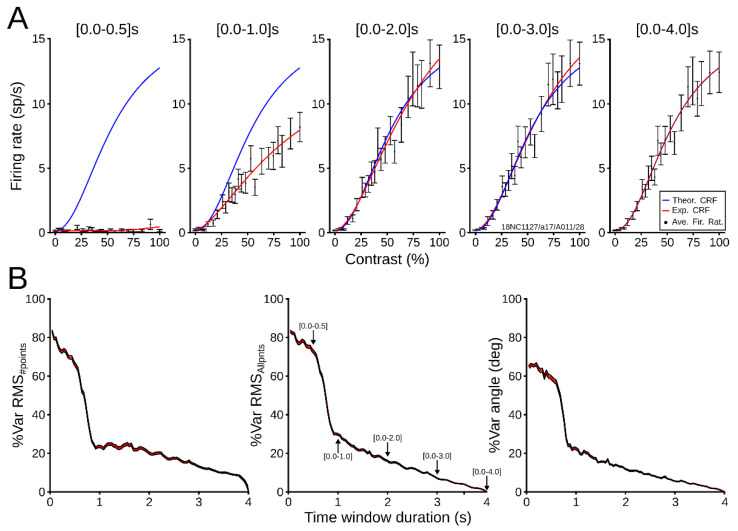
Dynamic response of CRFs. (**A**) Example of CRF fit to increasing lengths of recording time (red line). Average firing rates (black dots) are drawn with SEM (error bars). The theoretical curve is also depicted (blue line). (**B**) Average percentage of variation of RMS_#points_, RMS_Allpnts,_ and angle difference between vectors as a function of increasing trail duration. Average (red line) and confidence interval of the mean (black lines) were obtained by bootstrapping the data (1000 iterations).

To explore how CRFs vary across time, we quantified errors dynamically along with the recordings. The CRF for each neuron was calculated with a window that gradually enlarges until it covers the entire registration period. Figure 9A shows a representative case of this adaptation analysis, in which the experimental CRF (red line) is compared to the theoretical fit (blue line). For short quantification periods, the experimental fit was very different from the theoretical CRF, mainly because short periods collected insufficient spike data to construct a similar curve to that of the theoretical CRF. When the windows were 1 s long, the dynamic range of the tested CRF increased, but its maximum firing rate was smaller than that of the theoretical curve. As recording periods were longer than two seconds, the tested curve was similar to the theoretical curve.

Mean neuronal profiles were further investigated by calculating the dynamic changes of the average RMS_#points_, RMS_Allpnts_, and angle vector difference. As Figure 9B shows, the average percentage of error variations was computed as the temporal window increased progressively. In general, errors were high, larger than 50% of the variation, just before 1 s of trial duration. After this point, average errors dropped around 30%. For a window of 2 s, errors were around 20% of the variation. Then, values gradually decreased as the window temporally increased to four seconds, where experimental and theoretical curves were equal. For RMS_#points_, the drop was much steeper than for the other two errors. Taken together, CRFs had lower errors close to one-second recording, and curves stabilized further from two seconds to end time.

## 4. Discussion

This study presents experimental and theoretical procedures to enable a reliable CRF that fits extensive experimental data in a reasonable recording period. With theoretical simulations, first, we found that changing parameters of the NRE varied little in CRF fitting. On the contrary, CRF fitting improved when experimental conditions (i.e., #points, #trials, trial length) increased, in which the number of repetitions was the most significant variable. After simulating several patterns with different experimental conditions, we found that a trade-off between the fit performance and the time duration exists to find adequate CRFs. Trade-off points were tested then experimentally to search for patterns that satisfied our two requirements. Four sets of experimental conditions that best satisfied these two constraints were revealed: {6, 16, 2.0}, {6, 30, 1.0], {4, 12, 4.0}, and {4, 20, 2.0}, meaning that with six or less contrast percentage points a sufficiently good curve fit can be recovered. Finally, we identified that recordings closer and longer to 1 s improved the CRF fitting than shorter periods. Our results suggest that their implementation in online recordings that require simultaneous sampling of numerous neurons will improve the adjustment of CRF fitting using short periods.

The pattern had the smallest average error among the four sets of experimental conditions, so the best performance was {6, 16, 2.0} (Figure 7). Depending on the error used, only the pattern {6, 30, 1.0} showed significant differences with it. That pattern {6, 16, 2.0} is better than pattern {6, 30, 1.0} would be consistent with our previous results of optimizing the fit by a high number of repetitions and an acquisition period equal to or longer than 1 s. Another way to choose between patterns is to rank them by the shortest recording time generated: {4, 20, 2.0} < {6, 30, 1.0} < {6, 16, 2.0} = {4, 12, 4.0}. In this case, pattern {4, 20, 2.0} will be enough to infer the best fitting curve as it has already been experimentally implemented [21]. However, depending on the scale used, CRFs formed with four contrast points can seriously damage contrast curve fitting. For example, scale 8 (Figure 7A,B), in which points are distributed towards contrast boundaries (0% and 100%), induces a poor quality curve fit since the central zone remains unexplored and exposed to high fluctuations. Then, the pattern {4, 12, 4.0} should be applied to fit the contrast curve, but its experimental duration is equal to that of the {6, 16, 2.0} pattern.

The fluctuations observed for the scales were even more pronounced when three extreme cases of CRF were investigated. For all 3 cases, it is quantified that scales 2, 3, 6, and 8 perform poorly when using RMS_Allpnts_, and even worse when using RMS_#points_. We highlight that scales 2, 3, and 6 should be avoided when fitting CRFs with few contrast points. In general, the effect was different when the angle was used as an error, where scales 3 and 6 had poor optimization, and scales 2 and 5 had high performance. Interestingly, there is a trend in the angle error for the patterns that performed better. This result shows that CRFs that tend to be more linear optimize better with more repetition trials (C_50_ = 100%), whereas nonlinear curves (n > 3.5) are more likely to improve the fit as the number of contrast sampled increases. This tendency would be effective for scales with explicit contrasts at 0% and 100%. Thus, in the case of CRFs with a linear tendency, such as curves from functional magnetic resonance imaging (fMRI) [22,23], the number of repetitions should be prioritized for a good fit, while curves that tend to be more nonlinear, such as those from an electroencephalogram (EEG) [24,25,26], should prioritize the number of contrasts sampled.

One caveat of the theoretical model is the lack of neuronal adaptation. Neuronal adaptation can act as a band-pass temporal filter to affect spike responses to subsequently presented stimuli for tens to hundreds of milliseconds [18,27]. We avoid neuronal phenomena by imposing an interstimulus interval (a blank grey screen between different contrast stimuli). Another problem of neural adaptation is that long acquisition periods would have been favored over patterns with shorter periods. Therefore, patterns with shorter acquisition times could be less often imposed. In the theoretical sections, 35.8% of the patterns selected to be then tested experimentally had 1 s of duration. In the experimental validation, besides {6, 30, 1.0}, another pattern that appeared ranked (repeated twice between errors) as a solution having short recording time was {4, 40, 1.0}. Other patterns that ranked lower (only appearing in one type of error) as possible solutions were {8, 10, 1.0}, {4, 30, 1.0}, {8, 20, 1}, {6, 20, 1.0}, and {12, 16, 1.0} (Figure 6). Therefore, 43% of the best-ranked patterns were for periods of 1 s. These findings suggest that short recordings, where adaptation is less pronounced, were often selected. We found that a period of one second decreased the variation of the errors substantially (Figure 9). The only requirement to stabilize CRF formed by one-second recordings should be to increase the number of trials (Figure 4C).

Three types of errors were used in this work. Their implementation was to ensure an objective comparison between contrast data points and the theoretical curve (RMS_#points_), between the two curves (RMS_Allpnts_), and the NRE parameters (angle between two vectors). As shown in Figure 5 and, later in detail for best patterns, Figure 6, the performance of the scales is highly dependent on the type of error used. For example, the logarithmic scale 2, which is widely used [27,28,29,30,31,32], shows a high error value for RMS_#points_, lower for RMS_Allpnts_, and low and nonsignificant for the angle. This variation suggests that the logarithmic scale is poorly estimated by calculating a few points of comparison between the theoretical and experimental curves. This comparison would suggest that error estimations that show few contrast points would not be as robust as those that sample the entire curve [33]. Using other scales or other estimators is preferable, such as the angle between NRE parameters. A further comparison of errors used here with another estimator, the χ2, revealed that outputs are more similar to the angle values than the RMS type errors (Supplementary Figure S3). This analysis may validate the better estimation of errors by calculating the NRE parameters rather than by comparing data points of the curve. Although differences between the solutions of each error are observed, the experimental conditions show a convergence between their solutions. Four solutions are repeated among the errors, suggesting a consistency of the analysis performed in this study.

As scale 6 shows, CRFs without the contrast at 0% performed poorly. For this scale, the baseline, B, is estimated from contrast points bigger than zero. Even if the literature shows that it is pretty common to use it [34,35,36,37], Figure 5 and Figure 6 show that the lack of contrast = 0% causes fit failures. As such, we suggest considering the use of a dynamic range involving contrast = 0% for fitting CRFs.

## 5. Conclusions

Four sets of experimental conditions satisfy the trade-off between fit performance and time of recording: {6, 16, 2.0}, {6, 30, 1.0}, {4, 12, 4.0}, and {4, 20, 2.0}. These sets have to include the contrast point at 0. A recording period closer and longer to 1 s is needed to obtain satisfactory CRF fittings.

## Figures and Tables

**Table 1:** Functional and experimental parameter values that were varied in the Monte Carlo simulations (all simulations).

	CRF Parameter	Values for Simulations	Units
Functional	*R*_*max*_	5, 7, 10, 16, 32	spk/s
	C_50_	20, 40, 50, 60, 80	%
	*B*	1, 2, 4	spk/s
	*n*	1, 2, 3, 6	-
Experimental	Trial Length	1, 2, 4, 6, 8, 16	s
	# contrast points	4, 6, 8, 10, 15, 20	-
	# repetitions	1, 2, 4, 8, 16, 32, 64	-

**Table 2:** Metrics used during theoretical and experimental tests. For a given number of points *T*, we have the following 10 metrics as depicted in Figure 3.

	Implementation			
	Scale	Lower Bound	Upper Bound	Description
1	Linear	0.0	1.0	Linearly spaced
2	Logarithmic	−1.2	0.0	Concentrated around 0
3	Logarithmic	−1.0	−0.15	Concentrated around 0.25
4	Logarithmic	−0.3	0.0	Concentrated around 1
5	Logarithmic	−0.7	0.0	Concentrated around 0.75
* 6	Logarithmic	−0.5	0.0	Same as 5 without 0
7	Logarithmic	−0.5	−0.15	Concentrated around 0.5
8	Linear	0.1	0.9	Same as 7 but linearly spaced
9	Linear	0.25	0.75	Same as 8 but less spread out
10	Logarithmic	−0.7	−0.1	Log-concentrated around 0.5

* Note that for metric spacing 6, there is no point at *c* = 0, therefore the baseline, *B*, parameter has to be estimated from the rest of the points.

## Data Availability

The data that support the findings of this study are available from the corresponding author, NC, upon reasonable request.

## Supplementary Materials

The following supporting information can be downloaded at: https://www.mdpi.com/article/10.3390/vision6040062/s1, Figure S1: Determining optimal least-square curve-fitting boundaries on *R_max_* and *n* (slope), Figure S2: Correlation between parameters of CRFs obtained from experimental neurons, Figure S3: Analysis of *χ*^2^; Table S1: Boundaries tested for CRF parameter *n*, Table S2: Boundaries tested for CRF parameter *R_max_*, Table S3: Comparison of scales for average output of the three errors used in Figure 5E, Table S4: Comparison of scales of the three errors used in Figure 7, Table S5: ANOVA for scales output of the three errors used in Figure 7.
