# Supplementary material for: Reliable, Fast and Stable Contrast Response Function Estimation
Source: Vision (Basel). 2022 Oct 17;6(4):62. doi: 10.3390/vision6040062 (PMC9589942; doi:10.3390/vision6040062)
Supplement: Supplementary file 1 [file vision-06-00062-s001.zip › vision-1772911-supplementary.pdf]

Supplementary Material

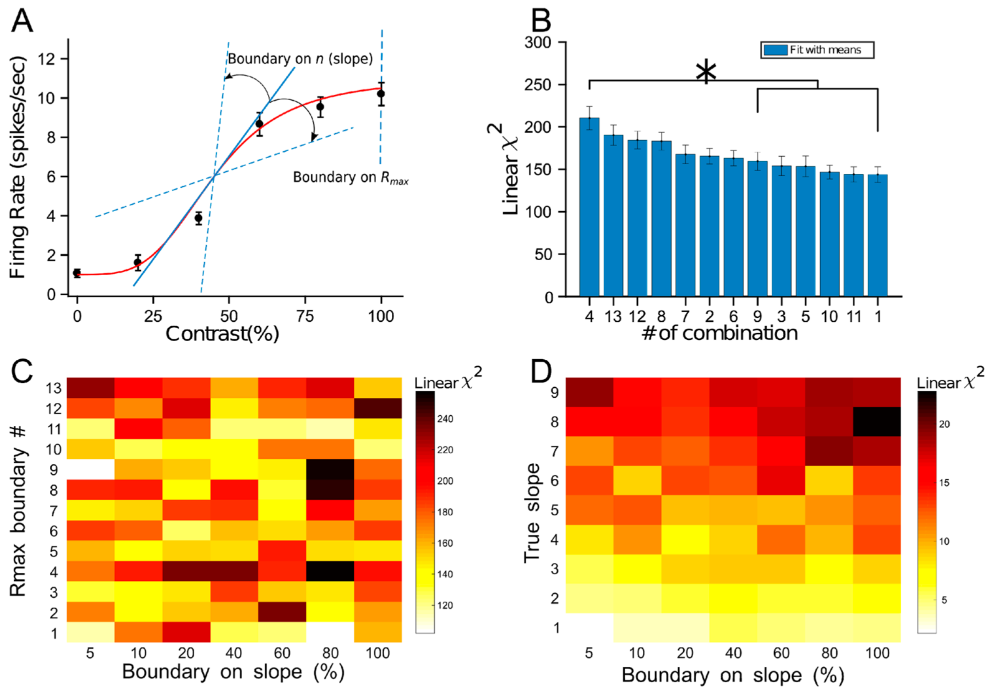

Figure S1: Determining optimal least-square curve-fitting boundaries on R_{max} and n (slope). A) Illustration of optimal boundaries on R_{max} and slope parameter n of the CRF. B) Optimal boundary combination for R_{max}. C) Influence of both parameter boundaries on fit error. D) Boundaries on n for all "true" n values.

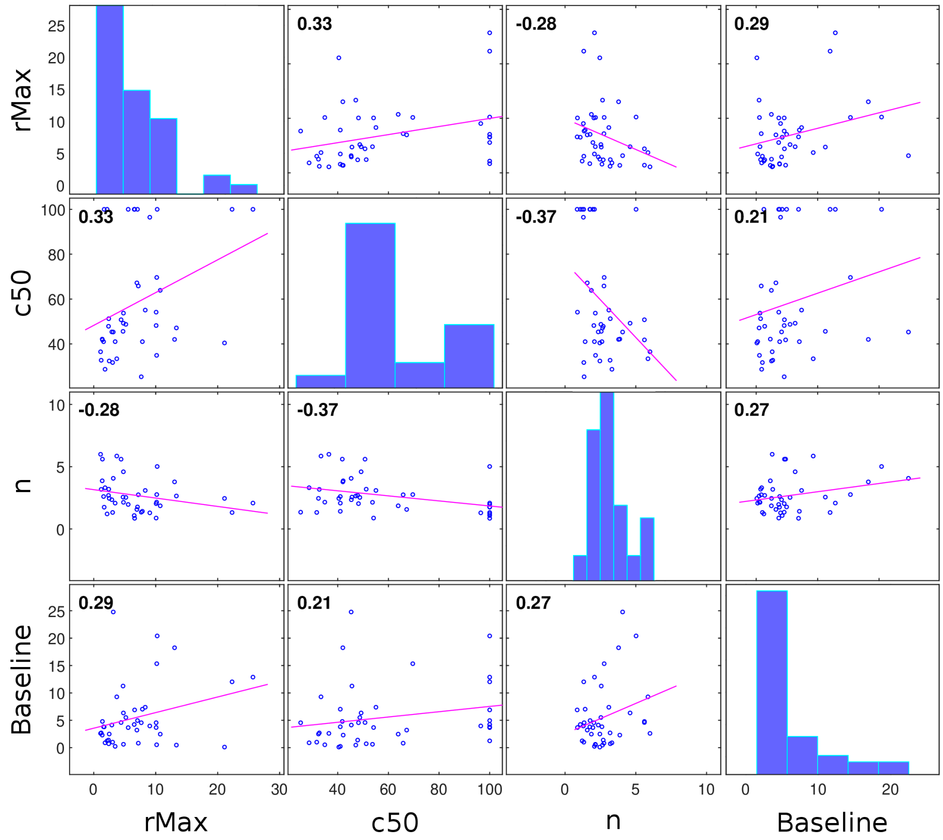

Figure S2: Correlation between parameters of CRFs obtained from experimental neurons. Pearson's correlation coefficients are described in the upper left corner of the graphics. Histograms of parameters are shown in the central element of the matrix.

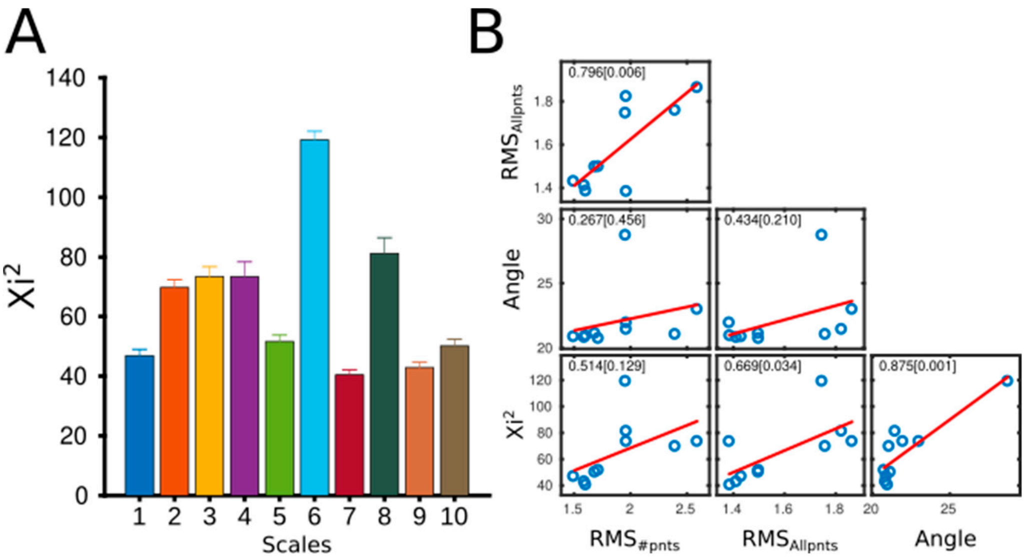

Figure S3: Analysis of χ^2. A) Average χ^2 across scales. B) Pearson's correlation coefficients between errors of scales, including χ^2. At the top of each figure, the correlation coefficient and its associated (p-value).

	**n boundary factor**
1	1.05
2	1.1
3	1.2
4	1.4
5	1.6
6	1.8
7	2.0

Table S1: Boundaries tested for CRF parameter *n*. The values are indicated as factors of the "true" n value.

	Rmax boundary
1	MAX
2	MAX-MIN
3	LAST
4	LAST-FIRST
5	MAX-MAXERR
6	MAX-MIN+MAXERR
7	LAST+MAXERR
8	LAST-FIRST+MAXERR
9	2 * MAX
10	2 * (MAX-MIN)
11	2 * LAST
12	2 * (LAST-FIRST)
13	No boundary

Table S2: Boundaries tested for CRF parameter R_{max}. MAX: the maximum firing response to contrast. MIN: the minimum firing response to contrast. LAST: the response to contrast at the last point (closest to 100% contrast). FIRST: the response to contrast at the first point (closest to 0% contrast). MAXERR: the maximum uncertainty (2 standard deviations) of the maximum firing rate.

Scale		p-value RMS#points	p-value RMSAllpnts	p-value angle(deg)
1	2	**0.000**	**0.000**	1.000
1	3	**0.000**	**0.000**	**0.000**
1	4	**0.000**	0.998	**0.023**
1	5	**0.009**	0.974	1.000
1	6	**0.000**	**0.000**	**0.000**
1	7	0.725	0.999	1.000
1	8	**0.000**	**0.000**	0.730
1	9	0.848	1.000	1.000
1	10	0.050	0.976	0.999
2	3	**0.034**	0.712	**0.000**
2	4	**0.000**	**0.000**	0.125
2	5	**0.000**	**0.000**	0.992
2	6	**0.000**	1.000	**0.000**
2	7	**0.000**	**0.000**	1.000
2	8	**0.000**	0.983	0.967
2	9	**0.000**	**0.000**	0.999
2	10	**0.000**	**0.000**	1.000
3	4	**0.000**	**0.000**	**0.032**
3	5	**0.000**	**0.000**	**0.000**
3	6	**0.000**	0.556	**0.000**
3	7	**0.000**	**0.000**	**0.000**
3	8	**0.000**	0.999	**0.000**
3	9	**0.000**	**0.000**	**0.000**
3	10	**0.000**	**0.000**	**0.000**
4	5	**0.001**	0.593	**0.005**
4	6	1.000	**0.000**	**0.000**
4	7	**0.000**	1.000	0.052
4	8	1.000	**0.000**	0.849
4	9	**0.000**	1.000	**0.012**
4	10	**0.000**	0.604	0.216
5	6	**0.002**	**0.001**	**0.000**
5	7	0.696	0.618	1.000
5	8	**0.001**	**0.000**	0.423
5	9	0.544	0.883	1.000
5	10	1.000	1.000	0.965
6	7	**0.000**	**0.000**	**0.000**
6	8	1.000	0.944	**0.000**
6	9	**0.000**	**0.000**	**0.000**
6	10	**0.000**	**0.001**	**0.000**
7	8	**0.000**	**0.000**	0.871
7	9	1.000	1.000	1.000
7	10	0.943	0.628	1.000
8	9	**0.000**	**0.000**	0.604
8	10	**0.000**	**0.000**	0.992
9	10	0.866	0.890	0.993

Table S3: Comparison of scales for average output of the three errors used in Figure 5E.

Scale		Figure 6A1 RMS#points				Figure 6B1 RMSAllpnts		Figure 6C1 angle(deg)			
		[6 , 16, 2.0]	[6 , 30, 1.0]	[4 ,12, 4.0]	[4,20,4.0]	[4 ,12, 4.0]	[4,20,4.0]	[6 , 16, 2.0]	[6 , 30, 1.0]	[4 ,12, 4.0]	[4,20,4.0]
1	2	**0.000**	0.173	**0.000**	**0.005**	0.741	1.000	1.000	1.000	0.997	0.998
1	3	**0.000**	0.360	**0.000**	**0.000**	0.676	0.985	0.983	0.999	0.908	0.984
1	4	**0.034**	0.726	0.839	0.972	1.000	1.000	1.000	0.966	0.986	0.998
1	5	0.674	1.000	0.324	0.713	0.947	1.000	1.000	1.000	1.000	1.000
1	6	0.750	0.836	0.517	0.983	0.719	0.971	**0.001**	**0.004**	**0.000**	**0.001**
1	7	0.982	1.000	1.000	1.000	1.000	1.000	1.000	1.000	1.000	1.000
1	8	0.997	1.000	**0.000**	**0.000**	0.000	0.000	1.000	1.000	0.747	0.684
1	9	1.000	1.000	0.993	1.000	1.000	1.000	1.000	1.000	1.000	1.000
1	10	0.999	1.000	0.509	0.914	0.899	0.998	1.000	1.000	1.000	1.000
2	3	1.000	1.000	0.999	0.962	1.000	1.000	0.950	0.995	1.000	1.000
2	4	0.714	0.997	0.070	0.196	0.844	0.998	1.000	0.926	1.000	1.000
2	5	**0.041**	0.290	0.410	0.586	1.000	1.000	1.000	1.000	0.951	0.976
2	6	**0.029**	0.987	0.239	0.159	1.000	1.000	**0.001**	**0.002**	**0.000**	**0.018**
2	7	**0.003**	0.334	**0.000**	0.002	0.819	0.999	1.000	1.000	1.000	1.000
2	8	**0.001**	0.412	0.970	0.780	**0.000**	**0.000**	1.000	1.000	0.997	0.988
2	9	**0.000**	0.232	**0.009**	0.030	0.968	1.000	1.000	1.000	0.994	0.999
2	10	**0.001**	0.167	0.245	0.319	1.000	1.000	1.000	1.000	1.000	1.000
3	4	0.274	1.000	**0.006**	0.004	0.791	0.938	1.000	1.000	1.000	1.000
3	5	**0.004**	0.524	0.082	0.035	1.000	1.000	0.991	1.000	0.678	0.900
3	6	**0.003**	0.999	**0.035**	0.003	1.000	1.000	0.068	0.054	**0.002**	**0.048**
3	7	**0.000**	0.577	**0.000**	0.000	0.763	0.947	0.936	0.983	0.983	0.999
3	8	**0.000**	0.663	1.000	1.000	**0.000**	**0.000**	0.951	1.000	1.000	0.999
3	9	**0.000**	0.448	**0.000**	0.000	0.949	0.996	0.904	0.990	0.874	0.990
3	10	**0.000**	0.350	**0.036**	0.009	1.000	1.000	0.987	0.999	0.974	1.000
4	5	0.930	0.861	0.999	1.000	0.980	0.999	1.000	0.999	0.884	0.973
4	6	0.890	1.000	1.000	1.000	0.826	0.902	**0.008**	0.186	**0.000**	**0.020**
4	7	0.488	0.893	0.865	0.920	1.000	1.000	0.999	0.855	0.999	1.000
4	8	0.320	0.934	0.001	0.001	**0.000**	**0.000**	1.000	0.988	1.000	0.989
4	9	0.076	0.806	1.000	1.000	1.000	1.000	0.998	0.889	0.976	0.999
4	10	0.245	0.716	1.000	1.000	0.953	0.987	1.000	0.971	0.998	1.000
5	6	1.000	0.932	1.000	0.999	1.000	1.000	**0.002**	**0.022**	**0.000**	**0.000**
5	7	0.999	1.000	0.358	0.560	0.973	0.999	1.000	0.998	0.999	0.999
5	8	0.991	1.000	**0.018**	0.007	**0.000**	**0.000**	1.000	1.000	0.450	0.426
5	9	0.835	1.000	0.922	0.953	0.999	1.000	1.000	0.999	1.000	1.000
5	10	0.979	1.000	1.000	1.000	1.000	1.000	1.000	1.000	1.000	0.994
6	7	1.000	0.951	0.557	0.945	0.800	0.914	**0.000**	**0.001**	**0.000**	**0.004**
6	8	0.997	0.974	**0.006**	0.000	**0.000**	**0.000**	**0.001**	**0.007**	**0.005**	0.319
6	9	0.888	0.895	0.982	1.000	0.962	0.991	**0.000**	**0.001**	**0.000**	**0.001**
6	10	0.990	0.828	1.000	1.000	1.000	1.000	**0.002**	**0.004**	**0.000**	**0.009**
7	8	1.000	1.000	0.000	0.000	0.000	0.000	1.000	1.000	0.915	0.896
7	9	0.997	1.000	0.996	0.999	1.000	1.000	1.000	1.000	1.000	1.000
7	10	1.000	1.000	0.548	0.817	0.942	0.989	1.000	1.000	1.000	1.000

8	9	1.000	1.000	**0.000**	0.000	**0.000**	**0.000**	1.000	1.000	0.691	0.737
8	10	1.000	1.000	**0.007**	0.001	**0.000**	**0.000**	1.000	1.000	0.886	0.961
9	10	1.000	1.000	0.980	0.996	0.996	1.000	1.000	1.000	1.000	1.000

Table S4: Comparison of scales of the three errors used in Figure 7.

	[6 , 16, 2.0]	[6 , 30, 1.0]	[4 ,12, 4.0]	[4,20,4.0]	
F(1,9)	**8.05**	**2.2**	**8.93**	**8.62**	RMS#points
P-value	**5.21E-11**	**0.0214**	**2.52E-12**	**7.26E-12**	
F(1,9)	1.55	0.6	**7.25**	**6.75**	RMSAllpnts
P-value	0.1259	0.7943	**8.23E-10**	**4.66E-09**	
F(1,9)	**3.7335**	**3.2245**	**6.0057**	**3.531**	angle(deg)
P-value	**1.59E-04**	**8.57E-04**	**6.40E-08**	**3.12E-04**	

Table S5: ANOVA for scales output of the three errors used in Figure 7.

For that purpose, the χ^2 statistical test was used as an error estimator. It can be described as:

$$\chi^2 = \sum_{i=1}^{n} \frac{(E_i - T_i)^2}{T_i} \quad \text{, (S1)}$$

where n is the total number of i points on the CRF, E_i is the "experimental" data, and T_i is the theoretical curve. We choose to discretize both the theoretical and the tested functions into n = 100 points to evaluate the χ^2 coefficient. Here it must be emphasized that the χ^2 was used to estimate errors between curves, and not for assessing statistical significance. Therefore, χ^2 was named as error (or percentage of error) in the graphs.
